# An exploratory qualitative study of inter-agency health and social service partnerships focused on Aboriginal and Torres Strait Islander clients

**DOI:** 10.1186/s12913-024-11656-y

**Published:** 2024-12-18

**Authors:** Anna P. Dawson, Eugene Warrior, Odette Pearson, Mark Boyd, Judith Dwyer, Kim Morey, Tina Brodie, Kurt Towers, Sonia Waters, Cynthia Avila, Courtney Hammond, Katherine Lake, Uncle Frank Lampard, Uncle Frank Wanganeen, Olive Bennell, Darrien Bromley, Toni Shearing, Nathan Rigney, Schania Czygan, Nikki Clinch, Andrea Pitson, Alex Brown, Natasha J. Howard

**Affiliations:** 1 Wardliparingga Aboriginal Health Equity, South Australian Health and Research Institute, Kaurna Country, Adelaide, SA Australia; 2 Adelaide Medical School, The University of Adelaide, Kaurna Country, Adelaide, SA Australia; 3 Watto Purrunna Aboriginal Health Services, Northern Adelaide Local Health Network, Kaurna Country, Adelaide, SA Australia; 4 College of Medicine and Public Health, Flinders University, Kaurna Country, Adelaide, SA Australia; 5 Aboriginal Services, AnglicareSA, Kaurna Country, Adelaide, SA Australia; 6 Sonder, Kaurna Country, Adelaide, SA Australia; 7 Indigenous Health Equity, Melbourne School of Population and Global Health, The University of Melbourne, Wurundjeri Country, Carlton, VIC Australia; 8 Kaurna Elder and Aboriginal Community Representative, Kaurna Country, Adelaide, SA Australia; 9 Nunga Mi:Minar Inc, Kaurna Country, Adelaide, SA Australia; 10 InCompro Inc, Kaurna Country, Adelaide, SA Australia; 11 Aboriginal Health Promotion, Wellbeing SA, Kaurna Country, Adelaide, SA Australia; 12 South Australian Department for Corrections, Kaurna Country, Adelaide, SA Australia; 13 South Australian Department for Education, Kaurna Country, Adelaide, SA Australia; 14 Telethon Kids Institute, Kaurna Country, Adelaide, SA Australia; 15 Australian National University, Ngunnawal Country, Canberra, ACT Australia

**Keywords:** Aboriginal and Torres Strait Islander peoples, Inter-agency partnerships, Health services, Social services, Qualitative research

## Abstract

**Background:**

The siloed nature of the health and social service system threatens access for clients engaging numerous organisations. Many Aboriginal and Torres Strait Islander people face adverse circumstances which contribute to multiple health and social needs. Effective relationships between health and social services are integral to coordinated service provision to meet the diverse needs of Aboriginal and Torres Strait Islander clients. Place-specific insights into inter-agency relationships are needed to inform targeted strategies that bolster service coordination to benefit Aboriginal and Torres Strait Islander people.

**Methods:**

This study sought to understand experiences of inter-agency partnerships among health and social service providers on Kaurna Country in northern Adelaide using semi-structured interviews and yarning circles to explore partnership actions, outcomes, enablers, challenges, and identify strategies to strengthen partnerships. Fifty-nine service providers (78% female, 62% Aboriginal) participated including six from non-government organisations, 17 from Aboriginal community-controlled services and 36 from government organisations.

**Results:**

A content analysis identified partnership actions such as client advocacy, referrals, sharing information, case management meetings and collaborative tender submissions which were seen to improve client access, navigation and outcomes and strengthen worker connectedness and job satisfaction. Motivated workers, listening to Aboriginal people, shared goals and values, and partnership agreements (e.g., memorandum of understanding, service contracts) were identified enablers of partnerships. Racism and ignorance, lack of networking events, communication breakdown, red tape and administrative barriers, competition between services, short-term funding, high turnover of staff and a focus on key performance indicators rather than community needs were among the challenges. Effective partnerships to benefit Aboriginal communities in northern Adelaide was reported to require aligned intersectoral strategic intentions, reforms to service commissioning processes, sustainable funding, regular network events for management and frontline workforce, Aboriginal practitioner-led service coordination approaches and a network of Aboriginal and Torres Strait Islander workers across organisations.

**Conclusions:**

This study identified key leverage points for action on inter-agency partnerships to benefit Aboriginal and Torres Strait Islander communities on Kaurna Country. System drivers such as funded inter-agency networks and reforms to commissioning of services must support organisational- and practitioner-level enablers to strengthen partnerships between health and social services across northern Adelaide.

**Supplementary Information:**

The online version contains supplementary material available at 10.1186/s12913-024-11656-y.

## Background

Aboriginal and Torres Strait Islander peoples collectively represent the longest continuing cultures on the planet, having thrived on the Australian continent for countless generations and tens of thousands of years [1, 2]. The ongoing harms of colonisation following European invasion less than three centuries ago shape socio-economic experiences and health outcomes for Aboriginal and Torres Strait Islander people [3]. Pervasive unmet health and social needs necessitate frequent interactions with multiple services and clinicians at any one time which can be challenging for Aboriginal and Torres Strait Islander people who have had traumatic experiences in health and social services [4, 5]. As Aboriginal and Torres Strait Islander people attempt to navigate complex service systems there are many challenges such as racism and discrimination, confusing eligibility criteria, lack of access to technology and transport, and service providers who don’t understand or respect culture or listen to needs [4].

It is imperative that health and social services work collaboratively to coordinate the services provided to Aboriginal and Torres Strait Islander clients, to facilitate access and navigation across services, to provide culturally safe and respectful models of care, to demystify the complexities of eligibility and funding models, and to avoid duplication and over-servicing of clients while ensuring clients never fall through the gaps of care [4]. As Riggs and colleagues [6] highlight, ‘Inter-agency partnerships are critical for addressing the interrelated circumstances associated with the social and health determinants of health inequalities’ (p.780). While inter-agency partnerships across health and social service are essential to promote Aboriginal and Torres Strait Islander health, in the context of a colonial history there are additional considerations related to intercultural relationships and the organisations involved.

Health and social services for Aboriginal and Torres Strait Islander people are provided by government (GOV) and non-government organisations (NGOs), private businesses and Aboriginal community-controlled organisations (ACCOs) with additional programs coordinated by local Councils. Government services are funded by both Commonwealth and State governments (e.g., hospitals and primary health care services) while NGOs can be church-, community- or philanthropy-based not-for-profit organisations that provide services from their own resources or commissioned and funded by governments through service contracts. ACCOs are unique in that they are governed by an elected Board of Management comprising representatives from their local Aboriginal and Torres Strait Islander communities [7]. They are funded by Commonwealth and State governments, provide services targeted to the needs of their local communities, and commonly employ a majority Aboriginal and Torres Strait Islander workforce [7, 8]. ACCO primary health care services play a key role in advocacy and navigation of the mainstream service system for their clients and communities [9]. However, the investment required to develop and maintain partnerships with multiple external agencies is a recognised challenge facing the sector [8]. Compounding this challenge are power differentials in partnerships between ACCO and mainstream organisations resulting from unequal control and level of resources [10].

The northern region of Adelaide in South Australia falls within the top 25% of disadvantaged areas in Australia, according to the Index of Relative Socioeconomic Disadvantage [11]. Aboriginal and Torres Strait Islander people living here experience greater health inequity [12]. Aboriginal clients with mental health and substance challenges are described to be ‘getting the run around’ from a siloed service sector, with ‘frequent handballing’ across organisations leading clients to repeatedly ‘re-tell their comorbidity stories or cease looking for help until the next crisis’ (p.595) [13]. The Northern Adelaide Local Health Network (a state government agency) has a mandate to deliver primary, secondary and tertiary health care across the region, and identifies partnering as one of six strategic imperatives with a recognised need for ‘integrated health services across the continuum of care’ (p.18) [14]. This call for integration and partnership is a welcome response to the reported fragmentation of services in the catchment and the recognised gaps in core services for Aboriginal and Torres Strait Islander people [15].

It has been more than three decades since the National Aboriginal Health Strategy called for increased coordination across funding bodies, service providers and communities to promote the integration of health and essential services for Aboriginal and Torres Strait Islander peoples [16], with subsequent and current strategies reaffirming this necessity [17]. Partnership activities are not adequately funded by governments, however, and Aboriginal community-controlled health sector representatives identify greater recognition and resourcing of relationship building activities as an important and necessary policy reform [8]. Achieving effective and sustainable intersectoral partnerships can be difficult, with partnerships between ACCOs and mainstream health services (i.e., provided by non-Indigenous government organisations and NGOs) challenged by the legacy of Australia’s colonial history, different approaches to servicing clients, and insufficient resources [18]. Mistrust and tension, communication challenges, different ways of working, referral challenges and resource limitations are common concerns of employees across organisations [19]. Despite such challenges, many successful inter-agency partnerships exist with reported positive outcomes such as broadened service capacity and continuity of care [18]. Given that partnerships are ‘a fundamental part of any strategy for improving health outcomes for Aboriginal people’ (p.48) [20] we must strive to understand how to overcome the specific challenges impacting partnerships through tailored strategies that address both historical and contemporary concerns to promote sustainable intersectoral collaboration.

In a region where Aboriginal and Torres Strait Islander people require coordinated responses to their health and social needs, we sought to understand how to operationalise effective inter-agency partnerships. The aim of this study was to explore the nature of inter-agency partnerships across organisations that provide health and social services to Aboriginal and Torres Strait Islander peoples in northern Adelaide. Specifically, we sought to characterise partnership actions, outcomes, enablers, and challenges and to identify multi-level strategies and levers with perceived potential to strengthen inter-agency partnerships to benefit Aboriginal and Torres Strait Islander communities.

## Methods

### Study design

We undertook a descriptive qualitative study guided by Indigenous methodology principles. Our intent was to privilege local Indigenous voices [21] and knowledges [22]. We positioned local Aboriginal people working in service provision roles as ‘co-designers, co-implementers and co-knowledge translators’ [23] (p.72) of the research process through considered recruitment in research participant and research governance roles. We received ethical approval from relevant human research ethics committees and applied principles for Aboriginal health research co-created with Aboriginal and Torres Strait Islander communities across South Australia [24]. The research was undertaken with ethical approval from multiple jurisdictions. This study represented the second stage in a 5-year program of research titled *Taingiwilta Pirku Kawantila* (Strong Community in the North) that seeks to examine whether codesigned, tailored, multi-level strategies to optimise and coordinate the health and social service system can meet the needs of Aboriginal and Torres Strait Islander peoples and strengthen social and emotional wellbeing.

The research is undertaken on the traditional lands of the Kaurna people in the northern Adelaide region which has the greatest density of Aboriginal and Torres Strait Islander people in the metropolitan area [25]. The study region focuses on the catchment of the Northern Adelaide Local Health Network. The research was designed in response to research priorities identified by Aboriginal communities across South Australia [26] and was undertaken by the Wardliparingga Aboriginal Health Equity research group at the South Australian Health and Medical Research Institute in Adelaide. The study investigators include Aboriginal and Torres Strait Islander investigators working in a cross-cultural partnership with non-Indigenous investigators. An Aboriginal Governance Panel comprising Aboriginal Elders and staff living and/or working in northern Adelaide oversees the research and provides the research team with cultural and contextual guidance in relation to community engagement, data collection and interpretation, and meaningful translation of findings to achieve maximum benefit. The research team includes a respected local Aboriginal person in a Knowledge Broker role [27, 28] to connect the research team with the Aboriginal and Torres Strait Islander community and service providers in northern Adelaide.

### Participant recruitment and data collection

Recruitment to semi-structured interviews and yarning circles was purposive. The Knowledge Broker invited staff employed in health and social services’ organisations that provide services to Aboriginal and Torres Strait Islander people in northern Adelaide. Staff members were Aboriginal and/or Torres Strait Islander and non-Indigenous people and were employed in ACCOs, NGOs, government services and local Councils. They were provided with written information about the study and an opportunity to ask questions prior to providing informed consent. A Distress Protocol (Supplementary File 1) was also provided that outlined the supports available if distress was experienced during participation. Interviews and yarning circles were facilitated by the Knowledge Broker with another member of the research team present to take notes in most instances. The Knowledge Broker invited participants to share their perspectives on the ways organisations work together to deliver programs and services to the Aboriginal and Torres Strait Islander community. Specifically, participants were invited to share experiences of both effective and ineffective partnerships, the benefits of effective partnerships, the enablers and challenges of partnerships, the partnership strategies required to facilitate navigation of health and social service organisations and the strategies needed to strengthen integration between organisations to benefit Aboriginal and Torres Strait Islander clients (Supplementary File 2). Data gathering with Aboriginal and Torres Strait Islander participants incorporated yarning, an ‘Indigenous cultural form of conversation’ (p.37) [29]. Social yarning facilitated connections between participants and the research team, and research topic yarning was also welcomed. Cultural protocols were followed including acknowledging Country, introductions that connected those present to family and place, and pausing for a Minute of Silence to show respect to Elders and other community members who have passed away [30]. Two interviews were undertaken via videoconferencing and the remainder took place in person in workplace settings that afforded privacy. In most instances, interviews and yarning circles were digitally recorded with consent and transcribed verbatim in preparation for analysis. Notes were taken during two yarning circles as approval to digitally record had not been given in an institutional ethical approval process.

### Data analysis

Transcribed text and written notes were analysed in NVivo software (QSR International Pty Ltd) by an Indigenous and non-Indigenous member of the research team applying the coding methods of Saldana [31] (i.e., initial coding and structural coding) and content analysis approach of Graneheim and Lundman [32]. This method facilitated a detailed analysis of the manifest (face-value) content of participant perspectives in addition to an analysis of underlying interpretive themes observed across participant accounts. Several transcripts were open coded and after reviewing the codes a structure of categories was established [31] related to partnership actions and principles, outcomes, enablers, challenges and proposed partnership strengthening strategies. Coding progressed within this structure and as sub-categories were developed, the structure was regularly reviewed and refined to best reflect participant perspectives at the level of the system and organisational strategy, operations, and service delivery. The research team presented emerging findings at a series of Aboriginal Governance Panel meetings to seek feedback and clarification and to undertake a process of collective member checking. A thematic analysis identified common narratives threaded across the dataset in relation to challenges, enablers and partnership strengthening initiatives. These themes were seen to represent key leverage points for action on interagency partnerships to strengthen service system responses to benefit Aboriginal and Torres Strait Islander communities.

## Results

Fifty-nine service providers participated in seven interviews and fourteen yarning circles (*n* = 2–8 participants). They were predominantly female (*n* = 46, 78%) and more than half were Aboriginal (*n* = 36, 61%). Participants worked in a range of health and social services sectors (E.g. education, family services, housing, domestic violence, mental health) and were employed in government services (*n* = 35), ACCOs (*n* = 17), NGOs (*n* = 6) and at a local Council (*n* = 1). Table 1 provides a description of participants. In the sections that follow, we report on participant perceptions of partnership actions, partnership enablers and principles, partnership outcomes, partnership challenges, and proposed strategies to strengthen partnerships. The final section of the results presents a summary of the themes observed across the dataset. All of the findings are summarised in Table 2.

**Table 1:** Participant description

	Aboriginal and Torres Strait Islander service providers	Non-Indigenous service providers	Total
Female	25	21	46
Male	11	2	13
Total	36	23	59
*Employment Sector*			
Health	9	4	13
Education	9	0	9
Legal	5	1	6
Family Services	1	3	4
Disability	2	0	2
Housing	0	9	9
Homelessness	2	2	4
Police	2	2	4
Drug and Alcohol	1	0	1
Domestic Violence	1	0	1
Mental Health	3	0	3
Community Services	0	2	2
Human Services	1	0	1
**Total**	36	23	59
*Organisation*			
Government	2	3	5
Non-government Organisation	22	14	36
Aboriginal Community Controlled Organisation	12	5	17
Council	0	1	1
**Total**	36	23	59

**Table 2:** Summary of findings: categories, sub-categories and themes

	System elements	Organisational elements		
		Strategic	Operational	Service delivery
	*System-level factors that impact inter-agency partnerships*	*How organisations interact from a strategic perspective*	*How organisations interact at an operational level* *(non-client related)*	*How organisations interact in relation to clients and service delivery*
**Partnership actions**		Collaborative tender submissions; joint projects; advocating to partners regarding new policies; inter-agency service improvement discussions; hosting intersectoral meetings; sharing resources	Regular inter-agency meetings; inviting partners to lunches and to be guest speakers; sharing information about staff roles and programs; shared coordination of staff training and conferences	Case management meetings; sharing information; advocating with partners on behalf of clients; referring and connecting clients to partner organisations; supporting community events
**Enablers**	Long-term funding; audits and standards that promote organisational accountability to partnerships; government contracts with aligned key performance indicators (KPIs)	Inter-agency agreements; investing time in partnerships; common goals, shared agendas and agreed outcomes; codesigning services with partner organisations; consent procedures to share information across services; active monitoring and evaluation of partnerships	Workforce attending community events; inviting partners to events and meetings; setting clear expectations of partner organisations; effective communication (having an equal voice); active management of conflict including tackling challenges together	Motivated workers who drive partnerships and demand client outcomes; Aboriginal and Torres Strait Islander workforce who enhance cultural safety and connections; workforce holding joint positions across organisations; workforce continuity; workforce mentoring and succession planning
**Positive outcomes**		Strengthened organisational capacity through access to partner expertise; enhanced sharing of resources; increased scope of services	Enhanced voice for the Aboriginal community through inter-agency advocacy; enhanced worker connectedness and job satisfaction; increased knowledge of other services and programs	Positive client outcomes, effective referral processes, enhanced sharing of information, enhanced responsiveness, improved follow up of clients, strengthened client access and service navigation, increased service continuity, strengthened wrap around support for clients
		Increased cultural safety and respect across the service system		
**Challenges**	Insecure funding, limited resources and capacity; commissioning of services leading to competition; government contracts for integrated services that fail to align on KPIs; lack of sustained networks and networking events for frontline workforce and executives	Incongruence in values, priorities and ways of working; privacy protocols (difficulty gaining consents to share information); short term staff contracts	High staff turnover; poor communication between services and within services; staff unaware of what others services provide	Unclear delineation of care coordination responsibilities; unresponsive organisations unable to accept referrals due to workforce shortages
		Racism, ignorance and a lack of cultural understanding in partner services including use of deficit models Staff who practice in a culturally unsafe way		
**Strategies to strengthen partnership**	A central Coordination Agency; Aboriginal community hubs and service centres; a Service Registry; aligned strategies co-created by governments and service providers; reduced competition and enhanced specialisation of services; sustained Networks; increased funding and incentives for collaboration	Universal consents to share information between services; intersectoral workshops focused on strengthening partnerships; measures to monitor and evaluate partnerships; a network of Aboriginal workforce across organisations	Fair remuneration to promote workforce retention and protect corporate knowledge; management support to attend network meetings; workforce exchange programs to increase knowledge of partner services	Increased Aboriginal leadership in Navigator and Partnership Coordinator roles; more frequent case management meetings; joint home visitsIncreased Aboriginal leadership in Navigator and Partnership Coordinator roles; more frequent case management meetings; joint home visits
**Themes**	A competitive service system undermines collaboration, fails Aboriginal people and wastes resources			
	Reforms to funding and commissioning of services	The value of Aboriginal workforce and leadership		
		Networks strengthen communication and cooperation		

### Partnership actions

A broad range of effective partnerships were described across health and human services, and across government, NGO, ACCO and local Council settings. Participants described partnerships activities focused on client care, focused on strengthening relationships with other organisations, and that were strategic in nature. The client-focused partnership activities included case management meetings, sharing client information (with consent), advocating with partner organisations on behalf of clients, referring and connecting clients to partner organisations, and supporting community events. Some partnerships were well established, and others were responsive ad hoc arrangements set up for specific clients as described by this community services’ worker:

*So*,* we’re collaborating with the health services*,* particularly the [name of organisation] in the North. They’ve been amazing with getting the children and the mum in really expediently to get them seen to. I can’t speak highly enough for how quick they’ve worked with my client*,* a case practitioner*,* and a health worker’s in there to kind of get them seen to really quickly. (Aboriginal service provider, ACCO, domestic violence sector)*

Relationship-focused activities included scheduling regular morning coffee meetings between staff of partner organisations, inviting guest speakers from partner services, inviting staff from partner services to monthly Nunga lunches^1^, sharing information across services about staff roles and available programs, and sharing coordination of staff training and conferences.

The strategic partnership activities included collaborative tenders and submissions, joint projects, advocating with partners to inspire new policies, bringing partners together to discuss service improvement strategies, hosting intersectoral collaborative meetings that focus on influencing systems, and utilising resources from partner services (e.g., transport, premises).

*I spoke to the (organisational representatives) and they’ve now got an MOU with us. They’ve started doing soup kitchen and they do raggedy old clothing. But that’s another thing*,* that they have an op shop. But the op shop is also a place where my staff can go in and do outreach. (Aboriginal service provider, NGO, Homelessness sector)*

### Partnership challenges

While participants described examples of successful partnerships, they also reported a lack of inter-agency connection and cooperation across the region. We heard that “*the service connections out of the northern suburbs are so disjointed*” with organisations described as “*disconnected*”, “*fractured*” and “*working in silos*”. The factors reported to constrain the number of services an organisation partnered with included limited staff, particularly Aboriginal staff; time; capacity to take on clients; promotion of services; lack of networks; the availability of services to partner with; mistrust of other services; and questionable reputations. Participants described multilevel challenges that undermine effective inter-agency collaboration in northern Adelaide. As summarised in Table 2 and described in detail below, these relate to insecure funding, limited resources and capacity; commissioning processes that promote competition; racism; incongruence in values and ways of working; uncertainty around care coordination responsibilities; communication breakdown; and lack of sustained networks and privacy protocols.

#### Insecure funding, limited resources and capacity

Participants described instability in the service system in the form of insecure funding, funding “*roadblocks*”, and agencies that come and go. Some respondents reported an unequal distribution of resources across collaborating organisations:

*So really*,* and don’t get me wrong*,* I get on with these people really well*,* but when they do these big partnership grants it’s not about equal distribution of that resource. (Aboriginal service provider, ACCO, drug and alcohol service)*

Short term funding arrangements leading to high turnover of staff was a common challenge. Participants reported a lack of capacity in some organisations, where staff didn’t understand what partner services could or couldn’t provide for clients. They described unresponsive organisations that lacked the capacity to accept referrals due to workforce shortages.

*I just think*,* obviously staffing*,* staffing issues are huge. So people are not able to communicate in a timely fashion where you’re waiting for days to get responses and – because people are so snowed down. So probably that’s one of my biggest challenges*,* is not being responded to. (Yarning circle participant, ACCO, health sector)*

#### Commissioning of services leading to competition

Funding and commissioning processes that created competition between services were commonly described by participants. Red tape and administrative barriers were also identified. We consistently heard about the challenges of services that were focused on “*their own self-survival”*, competing for funding and clients.

*That’s the problem with services. It’s all about their own self-survival and we are put into a marketplace where we are competitive with each other. (Aboriginal service provider, NGO, homelessness sector)*

We heard that when funding lines changed, services that had been de-funded did not want to work with organisations that had been awarded the new contract. Participants reflected that Aboriginal and Torres Strait Islander clients are “*missing out*” when organisations failed to collaborate, and that competition led to a “*a waste of resources*”.

*I think it’s difficult because it’s a competitive market and people don’t think about the social outcomes for the people*,* they think about the money for themselves and the funding*,* and the competing for funding and*,* I think*,* that’s really sad because people are left unsupported and vulnerable because we can’t get our shit together. (Yarning circle participant, NGO, community services’ sector)*

#### The impact of racism

Racism, ignorance and a lack of cultural understanding were identified as key challenges to safe working environments and inter-agency partnerships. Participants described staff who practiced in a culturally unsafe way, and non-Aboriginal organisations who used deficit models in relation to Aboriginal clients.

*The level of racism and ignorance and bad behaviour and assumptions that white staff and managers have had has impacted on the ability for those people to remain in this organisation. (Aboriginal service provider, NGO, homelessness sector)*

*A non-Aboriginal person will be sitting there and she’ll say*,* okay we’re going to be doing a Welcome now and you look at her and you go*,* okay that’s just set the platform of how I’m going to interact with you because there’s a high level of ignorance here in regards to what an acknowledgement is and what a welcome is and what are the protocols around that. So*,* on the outset*,* there’s a level of ignorance that we then go*,* oh god you’re kidding me. We’re up against this still*,* in this day and age? (Yarning circle participant, ACCO, family services sector)*

#### Unclear delineation of care coordination responsibilities among providers

Some participants felt there was a lack of clarity around which organisation was responsible to coordinate client care in instances where numerous organisations were providing services. As this respondent described:

*One of the biggest questions that’s been had out North is that somebody said*,* I don’t know six or seven years ago*,* there’s a billion dollars being spent on social services in the North*,* and nothing’s changing and why is that the case? …you can see why*,* nobody’s got ownership of the client*,* nobody’s actually in charge. There’s no one can tell you who’s got that responsibility now. (Non-indigenous service provider, council, community services sector)*

A deficit in inter-agency coordination was also echoed by these service providers in the education and community services sectors:

*Yeah. That’s it. There’s plenty of money there. Plenty of resources. It’s who do you get to come in and coordinate that in a better way. (Aboriginal service provider, GOV, education sector)*

*I’ve never actually thought it’s a budget issue*,* to be perfectly honest*,* I think there’s enough money and services*,* it’s just that they haven’t worked out how to do it together. (Non-indigenous service provider, council, community services sector)*

#### Incongruence in values and ways of working

A lack of trust in partner services was commonly raised. One participant reported “*often butting heads*” with partner services due to different ways of working. Services that imposed their rules and regulations were described, and instances where misaligned values across partnering organisations negatively impacted service delivery:

*But we’ve also had timely response*,* assuming that the values align*,* get into practice*,* and it doesn’t… (you) get to the actual delivery and it has been a train wreck. (Non-indigenous service provider, council, community services sector)*

Working under different worldviews, key performance indicators (KPIs), priorities, policies and procedures was another major inter-agency challenge.

*I think where sometimes it goes astray is that because the governments often – we’ll have a contract on the housing*,* [name or organisation] might have a contract on the support service*,* sometimes*,* they’re not exactly married together. So that can lead not to conflict*,* but just to different priorities in terms of*,* well*,* that’s where my money comes from*,* so that’s what I’ve got to do; and this is where my money comes – so that’s my priority. (Non-indigenous service provider, NGO, housing sector)*

There were concerns voiced related to organisations that inauthentically chased KPIs such as client interactions. Many services were criticised for being self-interested rather than focused on community needs.

*So we actually found that there was probably case coordinating sessions happening around individual clients*,* which would have eight services around one client and then they’re trying to map out the calendar and when to get a piece of him. Not even to go by the referral process or an assessment process*,* but just so each of the services can have a crack at the cherry to get some time to get a stat line down. So there was no Aboriginal lead to help the case coordination process*,* as a partnerships coordinator across the service sector. (Aboriginal service provider, GOV, human services sector)*

#### Lack of sustained networks for frontline workforce and executives

Lack of networking events for workforce was another common concern. Surprisingly, we discovered that many previous network attempts had failed and there was no formal, ongoing, recognised and supported network of Aboriginal and Torres Strait Islander workforce in northern Adelaide.

*Yeah. Been through processes where we’ve had meetings set up and then it’s been hard to continue those meetings*,* like those network meetings. So hopefully we can set them up again. (Yarning circle participant, GOV, health sector)*

*They’ve attempted several different types haven’t they*,* over the years … but they don’t last very long. They don’t keep going. (Yarning circle participant, ACCO, family services sector)*

#### Communication breakdown

Participants described poor communication both between services and within services. Time constraints were described as negatively impacting face to face meetings and communication with partner organisations. Participants reported poor internal communication in that they weren’t informed about their organisation’s strategy related to inter-agency partnerships and they didn’t know which other agencies their organisation had established formal relationships with.

Participants discussed the lack of communication between services as resulting in inefficiencies and lack of coordination across the service system.

*Participant: I’ve been in situations where some of our clients do go through other agencies and stuff*,* and you’re not even aware of it until you meet them for the fourth time*,* and you’re like*,* “Oh*,* okay*,* so you go through [name of organisation]?” That would’ve been great to know two months prior*,* because we could’ve connected up. There’s no connection between any services unless you are the one that is - - -*.

*Participant: Communicating.*

*Participant: - - - being the communicator and getting in contact with them. (Yarning circle participants, ACCO, legal sector)*

#### Privacy protocols

While sharing information was a key partnership action identified by participants, difficulties in gaining client consents to share information was a noted challenge.

*We don’t have a way to communicate easily*,* and everything requires a release of information and consents are one of the hardest things to get from our clients*,* so that’s a huge barrier. (Yarning Circle participant*,* NGO*,* Community Services sector)*

### Partnership enablers

Both system-level elements and organisational factors were reported by participants to strengthen and enable partnerships. The identified system-level enablers included long-term funding, audits and standards that promote organisational accountability to partnerships, and government contracts that have aligned KPIs.

*I do think government are getting better at this*,* I have to say*,* for more recent contracts I’ve seen them tender. I think there’s a much better understanding that they have to make sure that where you’re having more than one provider that their key performance indicators*,* all those sort of things are much better aligned so you don’t get that rubbing against each other a little bit. But I think that’s really key to making sure this is the intent. So you might be providing one part of it*,* and we’re providing the other*,* but the contract has got to make sure that the outcomes are really clear*,* how we’re going to measure is really clear. (Non-indigenous service provider, NGO, housing sector)*

The impact of tenders that call for partnerships and alliances was considered uncertain by some participants.

*And you see that with the current homelessness tender that’s out*,* which was a requirement for everyone to form alliances. They basically forced organisations to talk to each other. I’m not sure if it’s going to work*,* but that’s what they’ve forced people to do. (Non-Indigenous service provider*,* NGO*,* Housing sector)*

Community events and intersectoral network meetings were seen to enable partnerships and collaboration through strengthening interpersonal relationships and service responsiveness.

*So there used to be loads of network days and events and stuff*,* where all frontline workers would get together and work on projects or research of information*,* and that was great for people to get together and network*,* and actually get to know one another. You want a face to a name*,* then you’re going to be more likely to answer that phone call. (Yarning circle participant, NGO, community services sector)*

Formal inter-agency agreements and Memorandum of Understanding (MOUs) were seen to be strategic enablers by some participants, but others felt they were *“not worth the paper it’s written on”*, highlighting key people driving informal arrangements as integral to partnership success.

Facilitator: Do MOUs make a difference. Is it the formal or the - - -

*Participant: It’s the informal. It’s whoever is driving it makes a difference. MOU is not worth the paper it’s written on. It’s who’s driving it and the sad reality is*,* when the people who are driving it leave*,* it doesn’t happen anymore and that’s the problem.*

Facilitator: Because it’s based on key people and goodwill?

*Participant: Yeah*,* yeah. And if those key people are gone*,* then someone else might pick it up and run with it. But my experience*,* over a long period of time*,* is it’s the person who drives it. It’s not what’s on the paper. (Aboriginal service provider, NGO, homelessness sector)*

Other strategic enablers included common goals, shared agendas and agreed outcomes; organisational policies that promote engagement with partners; codesigning services with partner organisations; and active monitoring and evaluation of partnerships.

*So I think the beauty about some of those things is when you get together with likeminded organisations*,* you’ve got the same values*,* we’re trying to achieve the same things*,* it generally works pretty well. (Non-indigenous service provider, NGO, housing sector)*

*It is about compromise and it’s about pointing out what’s been good and what’s not been good and how can we change that? I’ve spent nearly all my day doing that*,* I think. (Aboriginal service provider*,* NGO*,* Homelessness sector)*

Operational enablers of partnerships included having consent procedures in place to share information across services and having the time to invest in partnerships. Motivated workers who drive partnerships and demand outcomes for clients was a common workforce-related enabler as described by this participant:

*My case manager was amazing at that. She would be like*,* “Hi*,* I’m from [name of organisation]*,* I’m going to bother you now. I’m going to keep your number and I’m going to be contacting you. Is that cool?” Like*,* super*,* ultra-friendly but that’s basically how she would go about it and she was really good at building connections so it always worked for her. It doesn’t always work for everybody else. (Yarning Circle participant*,* NGO*,* Community Services sector)*

Aboriginal and Torres Strait Islander workers were frequently described as enablers to partnerships since they enhanced cultural safety and trust across organisations and, through their connections to the Aboriginal and Torres Strait Islander community, facilitated linkages between services and clients. The importance of engaging with other services to provide "*warm referrals"* for clients was described, such as in this yarning circle with workers in a family services organisation:

*Participant: So if they’ve come in and they’ve been assessed and not fitting any of our services*,* we will then source another service to assist them. But we do a warm handover*,* so the client doesn’t have to retell their whole story. And we’re then supporting the client for however long it takes to work with a new service.*

*Participant: And a warm handover means you take them to that service*,* and you sit there*,* and you help them share their story*,* or you do it on their behalf*,* whatever they prefer.* *(Yarning circle participant*, *ACCO*, *Family services sector)*

Workers who moved on to another organisation within the same sector or who hold joint positions across organisations were seen to share knowledge of partner services and strengthen ties between services, respectively. Additional enablers included workers attending community events to connect with employees in partner services, continuity of workers, and mentoring and succession planning to promote the sustainability of partnerships.

*We also had that continuity of an Aboriginal project worker in [name of organisation]*, *and meeting that person earlier on*,* before they were going on to that program*,* that continuity of having that support of another Aboriginal person to follow through the services*,* from hospital to metro*,* to country*,* to home. It was just a really good flow for that patient. It was really good. (Yarning Circle participant, GOV, Health sector)*

*…a lot of us will move on*,* a lot of us grow and change and move. So it’s making sure that we pass on – so sort of like how our number system works*,* is passing on the information and making sure that the next person coming into this role is set up and successful. (Yarning circle participant*,* ACCO*,* Legal sector)*

Enablers at the relationship level included taking the time to actively build connections, inviting partners to events and meetings, being committed, and having clear expectations of partner organisations. Effective communication was consistently described as an enabler, and included clear communication around expectations, direct communication between CEOs, direction communication between frontline staff, and face-to-face meetings. It was considered important that partnering organisations have an equal voice, and that partners were able to tackle challenges and roadblocks together, to actively manage differences and conflict.

*I think it’s just being really clear about what you’re both trying to achieve and knowing the pathway is there and being open about*,* well*,* these are some of the challenges. I think one of the problems with our sector a little bit is often around money. The reality is there are certain things – things cost*,* and you’ve got to work out how you’re going to fund that. And I think some in our sector are just a little bit blind to that*,* just say*,* we’ll get more government money or whatever*,* but that isn’t how it happens. You’ve got to have a financial feasibility that actually works*,* and that’s not always easy to do. So*,* I think it’s acknowledging what the roadblocks are going to be for the outcome you’re trying to achieve*,* and how do you work on those roadblocks together and solve them? (Non-Indigenous service provider, NGO, Housing sector)*

### Partnership principles

The principles that were seen to enable and promote partnerships included trust, transparency, responsiveness, rapport, openness, mutual respect ("*I think the key thing is this mutual respect between the two organisations*"), integrity, flexibility, compromise, having an equal voice, accountability, professionalism, balancing mutual benefit and mutual obligation, and having aligned values in relation to client-centred care and self-determination. In one yarning circle, participants reflected on the importance of trust and the ability of organisations to adapt and be flexible to the needs of Aboriginal clients:

*Participant: And your client will tell you what organisations they will and won’t work with*,* and professionally*,* even there’s a couple of [name of organisations] that I won’t even let a client step foot in the door because I just don’t think that they do the right thing by community. So trust is a big thing.*

*Participant: Same. Yeah.*

*Participant: And it’s earnt and it’s easily lost.*

*Participant: Yeah*,* it’s hard to earn*,* easily lost.*

*Participant: And are they willing to*,* not bend the rules but not make life harder for a client who doesn’t meet 100% of the criteria. Like*,* what can you do to help? We know they’re not this*,* but what can you do to help out? Or how are you flexible to get that in? How are you going to engage with this person because this is their specific needs*,* you can’t pigeonhole them. Then how are you going to adapt to those needs? (Yarning Circle participants, NGO, Community Services sector)*

### Partnership outcomes

Participants described compelling benefits of partnerships in relation to positive client outcomes, effective referral processes, increased continuity in the service system through "*warm referrals"*, and enhanced sharing of resources.

*Just lots of little*,* the successes they can be huge from the way two organisations work collaboratively or collaboratively to effect really good outcomes for clients. To the littlest things*,* little steps that she just might have made in her life that seems so tiny to someone else but huge for our women. (Aboriginal service provider, ACCO, Domestic Violence sector)*

*My frontline workers don’t know what MOU we have with whoever it is*,* the relationships*,* so we’ve got an amazing relationship with a direct competitor which is [name or organisation]*,* we compete for the same skills*,* we live in the same space*,* we actually share a building in one space and I don’t know if we have an MOU or not*,* but our relationship is gold and we inter-refer*,* we refer in*,* we refer out*,* we share resources*,* we share carparks. (Yarning circle participant, NGO, Community Services sector)*

*Yeah*,* but for me success is not about what the organisation or the other partners that we have achieved. It’s about did we meet the need of the individual that we were trying to support*,* and they’re the stories that – it’s when we come together with a common agreed goal*,* and we achieve it on behalf of the client. And it’s their goal*,* not ours. (Aboriginal service provider*,* NGO*,* Homelessness sector)*

They also spoke of enhanced sharing of information across services, open doors, improved follow up of clients, increased knowledge of other services and programs, strengthened client access to services and navigation support, enhanced responsiveness of services, increased scope of services, and strengthened wrap around supports for clients. Some respondents spoke positively of the ability to ask favours of partner organisations:

*And just keeping those partnerships going as well because sometimes it will keep you – you can ask for favours*,* and we’ve got some pretty strong partnerships with other organisations that we’ve been able to ask favours if we’ve been stuck with maybe a trainer*,* to get some training done*,* and they can do it at a reduced price – us being not-for-profit*,* it also enables us to get things for maybe sometimes a little bit cheaper. (Aboriginal service provider, ACCO, Disability sector)*

The ability to tap into the expertise of partners was identified as a benefit for staff as it provided opportunities for learning and capacity building. Engaging in partnerships was seen to be important to provide voice to the community, as this Aboriginal service provider explained:

*So a lot of the times you get involved in partnerships because you know that it’s about providing the mob with some voice and hoping that you can direct whatever that partnership might be doing to the benefit of the mob*,* right. (Aboriginal service provider, ACCO, Drug and Alcohol service)*

Increased cultural safety and respect was another benefit, along with seeing new strategic actions in partner services resulting from advocacy activities. Service providers also spoke of effective partnerships leading to enhanced connectedness to workers in partner organisations and greater job satisfaction.

*For me*,* some of the benefit is knowing that we’re actually making a difference and doing a good job working*,* getting that feedback of*,* oh*,* well*,* that worked well*,* or such-and-such said*,* because I’ve got a good relationship with them*,* that maybe we should approach it this way. So you’re learning as well. Whereas*,* if you’re just trying to do it by yourself*,* and not having those partnerships*,* might blow up a little bit. (Yarning Circle participant, GOV, Health sector)*

Conversely, participants also described the negative outcomes of ineffective partnerships such as lack of information sharing, lack of connection to services, and clients and families falling through the gaps of care.

### Solutions to strengthen partnerships to benefit Aboriginal clients

A range of strategies were identified to strengthen partnerships between services in northern Adelaide and benefit Aboriginal and Torres Strait Islander communities. At the system level, participants strongly advocated for funded networks and regular networking events. They expressed that networks and network meetings were important not only for frontline service providers, but also for service executives:

*I don’t think it should be purely on a service-provider level either. I think there should be more integration at a higher level between services too. (Yarning circle participants, ACCO, Health sector)*

They proposed a register of services, and targeted workshops where service providers could reflect on how they can work better together.

*And think it would be really good to have a services workshop*,* where every service is out there talking*,* you know*,* and it has – not just the one day*,* like*,* a two day - about how we can work better together to support Aboriginal families and young people. (Aboriginal service provider*,* GOV*,* Education sector)*

Participants expressed that Aboriginal community hubs and service centres with co-located services would enhance inter-agency collaboration, and streamlined processes to share client information with consent would promote communication. Statewide strategic plans were considered important. Participants identified they should be "*led by government because they control the purse strings*" and cocreated with service organisations targeted to particular social needs (E.g. a housing strategy). Reducing competition between services to promote capacity strengthening in certain service provision areas was also proposed. Participants called for reduced duplication of services and increased specialisation.

*And*,* I think*,* if the government was smarter in how*,* like*,* [name of organisation] are great with homelessness*,* we’ve been working in that space for a long time so if the funding came to us for that*,* [name of organisation] are great with domestic violence and women*,* or [name of organisation]*,* is great for employment. If they’re actually distinct*,* if they didn’t make us compete for what we were good at and we were actually able to build our capacity in that area. (Yarning circle participant, NGO, Community Services sector)*

*And*,* partly*,* the system means that we do everything because all the businesses are pretty marginal. You’re always trying to grab a bit of business to keep things going and give yourself the opportunity to have a bit of growth. So I don’t know what I’m really saying. I suppose I’m saying I think people would be better served if there was more specialisation and fewer organisations within that specialisation. (Non-Indigenous service provider*,* NGO*,* Housing sector)*

Participants proposed other government-led initiatives such as incentivising collaboration and resourcing additional workers and a coordination agency whose role it is "*to ensure all other agencies are working together*". Increased staff remuneration was suggested to promote workforce retention and protect corporate knowledge. Seeking feedback from partners and establishing monitoring tools to ‘*measure whether you’re getting the right outcome’* were also identified as partnership strengthening strategies.

At the operational level, participants highlighted the importance of managerial support to attend network meetings. Aboriginal-led reforms to practice were proposed to strengthen service navigation, connections, and cultural safety across the service system. These reforms included roles for senior Aboriginal and Torres Strait Islander staff to interface with Aboriginal and Torres Strait Islander clients facing comorbid and complex health and social challenges to coordinate their services. These Aboriginal Coordinators would work with clients to determine needs, set goals, and bring in services in a staged approach based on these needs and goals.

*Yep and I think that their approach at the North was because services had disengaged with Aboriginal community*,* they said*,* let’s bring it all. Let’s just bring them all in and get the conversation going. But there was no case coordination*,* or there was no*,* and it probably needs to be an Aboriginal lead. Aboriginal lead to have the overarching say on who’s at the table*,* who’s not*,* for each individual client. And then to push them back into the services and go through the normal referral strings. But they need someone who can track it. So you probably need - if you’re not going to do a hub model*,* a one stop fits all*,* you need a partnership coordinator*,* a couple of them*,* floating across the different council districts… (Yarning Circle participant*,* GOV*,* Housing sector)*.

Time and trust were highlighted as integral to all future partnership strengthening strategies and accountable Aboriginal leaders and Elders were considered key. A workforce exchange program to enable frontline workers to learn about the functions of partner services was considered important, as was establishing a network of Aboriginal workers across partner organisations.

*So ideally having a little cohort of Aboriginal staff that our service could tap into within each of the organisations*,* do you know what I mean*,* like just having even one or two liaison workers within Centrelink or within Housing. So it’s a direct contact and we can call them. (Yarning circle participant*,* GOV*,* Health sector)*

Such a network was considered important to strengthen client navigation across the system. Other strategies to promote navigation included enhanced advocacy services and establishing a lead organisation to undertake case management and brokerage. Joint home visits and regular fortnightly meetings between service providers were also described.

### Thematic summary

We identified four themes in participant accounts, as included in Table 2. We observed that a fractured and competitive service system undermines collaboration, fails Aboriginal and Torres Strait Islander people and wastes resources. We consistently heard of services that were focused on "*their own self-survival*", competing for funding and clients and chasing KPIs rather than client outcomes. We heard about a lack of coordination, ineffective communication and of Aboriginal clients "*missing out*". Participants called for system level reforms to funding and commissioning of services to reduce duplication and competition, promote specialisation of services, incentivise cooperation and strengthen workforce continuity through secure funding. Among other strategies, they spoke of aligned key performance indicators in service contracts as having potential to synchronise inter-agency priorities and promote effective collaboration.

We heard that partnerships were undermined by racism, use of deficit models, and service providers that lacked cultural safety and failed to respect client-centred care. The value of frontline Aboriginal workers and leadership in making connections across services, in strengthening cultural safety across partnerships, and in connecting Aboriginal clients to partner services was frequently described. Aboriginal staff were seen to promote connections and strengthen trust across services. Specific navigator and partnership coordinator roles were proposed for Aboriginal staff. We observed that networks strengthen communication and cooperation and provide opportunities for staff to connect which is important in a system with high staff turnover. Networking activities for Aboriginal and Torres Strait Islander workforce at both frontline and managerial levels across the North was viewed as imperative. Networking events for workforce across services was considered vital, particularly when the events provided opportunities to actively reflect on how services can work better together.

## Discussion

Our study presents a detailed account of inter-agency partnership actions, outcomes, enablers, and challenges in northern Adelaide and identifies multi-level strategies with perceived potential to strengthen health and social service collaboration to benefit Aboriginal and Torres Strait Islander communities. Partnerships are heralded since they ‘achieve synergistic outcomes, which are more than can be achieved by individual partners or sectors working alone’ (p. 408) [33]. However, service providers engaged in this study shared the complexities facing service coordination in northern Adelaide and the instances where a lack of partnership and collaboration failed Aboriginal and Torres Strait Islander clients. They also identified enablers that support agencies to work together, the challenges that drove them apart, and provided examples of partnership successes where agencies effectively collaborated to support Aboriginal and Torres Strait Island clients. The fundamental question is this: how can the system and the service providers within it be structured to ensure that Aboriginal and Torres Strait Islander clients receive the right supports at the right time from culturally safe service providers that are focused on their needs and goals? Innovative strategies were proposed by service providers to reform service coordination and inter-sectoral partnerships to strengthen the responsiveness of the service sector for Aboriginal and Torres Strait Islander people. This study has identified the means through which some of this can be achieved, but there is further work to be done to bring governments and service providers together to reconsider the market model that drives competition rather than cooperation.

We heard that Aboriginal and Torres Strait Islander clients are missing out when services are ‘*put into a marketplace where we are competitive with each other*’. Examples were shared where agencies refused to work together because one service held the funding for a particular program only to lose it to the other service. Our findings raise questions about the validity of the competitive market model in Aboriginal and Torres Strait Islander health and social services. They highlight how competitive service provider behaviours motivated by this model undermine collaborative efforts to achieve outcomes for Aboriginal and Torres Strait Islander clients. Respondents described inefficiencies in the system due to duplication of services and proposed greater specialisation of services since ‘*people would be better served if there was more specialisation and fewer organisations within that specialisation’*. Participants highlighted that the system has been set up on a capital model where businesses are motivated to ‘*do everything’* because of marginal profits and the need to ‘*grab a bit of business to keep things going’*. But while agencies are chasing business, salaries are being spent on tender applications rather than on achieving outcomes for clients.

There were concerns raised about the nature of key performance indicators leading services to chase client ‘*stats’* to ‘*justify the funding’* rather than chase client goals to achieve client-centred outcomes. Our findings highlight that the performance indicators agencies are striving to achieve must be reconsidered since they focus on processes (e.g., numbers of clients and client interactions) rather than outcomes. Governments and service providers could come together to codesign such performance indicators. When the national key performance indicators for Aboriginal primary health care services were developed, they were imposed on the sector with tokenistic consultation and were seen as a funding requirement and accountability measure rather than a mechanism to strengthen quality [34]. This mistake must be avoided in efforts to establish performance indicators that promote service coordination for Aboriginal and Torres Strait Islander peoples.

Despite an existing evidence base describing challenges in northern Adelaide related to siloed services [13], and known trust challenges in intercultural partnerships [18, 19] this study has identified that the system still depends on motivated and ‘*passionate’* workers who ‘*drive’* partnerships to get results for their clients, rather than structural mechanisms for effective coordination. We heard that there is a need for both service executives and frontline employees to be more connected. We also heard that partnership agreements (e.g. MOUs) can help to secure relationships but are worthless without passionate people who actively strengthen the partnership. More work needs to be done to identify the mechanisms required to create and scaffold meaningful linkages and information flows across the service system. Participants identified that more must be done to promote active monitoring and evaluation of partnerships. This is supported by scoping review evidence which identified evaluation of partnerships for continuous improvement as one of nine core components of positive partnership processes [35]. Our data suggests that drivers of partnership improvement activities must be developed in much the same way as the standards for continuous quality improvement requisite for health service accreditation. These could be built in as additional key performance indicators in service contracts and funding agreements. Our study participants spoke of partnership actions that focused on strengthening inter-agency relationships as well as outward facing actions that strengthen partnerships through coordinated client care. Performance indicators could reflect this full gamut of partnership actions.

More than once we heard that beyond the need for additional and consistent funding, effective coordination of services is essential to ensure the needs of Aboriginal and Torres Strait Islander peoples are being met. We heard that system reform should include the employment of Aboriginal Coordinators who engage with clients to undertake a needs assessment, set goals and then coordinate service responses to navigate the service system. These Aboriginal and Torres Strait Islander workers would then connect their clients to other Aboriginal and Torres Strait Islander clinicians and to trustworthy non-Indigenous workers through ‘*warm handovers’*. The importance of intercultural connections across frontline workforce in addition to intercultural connections across organisations (government, NGO, ACCO and local Councils) is paramount [18, 19]. The necessity of connections across health and social services is clear [10]. Brodie and colleagues [36] piloted a model of social needs assessment, goal setting and brokerage with Aboriginal clients in a research setting, which is yet to be formally evaluated in a service setting. What is uncertain is where these Aboriginal Coordinators would be employed: in the health sector or the social services sector, and in Aboriginal community controlled or government organisations? ACCOs are known to strengthen cultural safety and connection to community [7, 37] and may be preferred by Aboriginal and Torres Strait Islander clients. Other innovative models of service provision could also be explored. Potentially, these Coordinators could be employed within Aboriginal and Torres Strait Islander community hubs or services centres to promote effective and coordinated care [4].

We found that passionate and committed workers were key to driving partnerships, and formal legal agreements were seen to enable partnerships in some instances. Fuller and colleagues (2005) found formal agreements along with common care-management tools and training were necessary to promote the sustainability of a collaborative mental health program. We heard consistent messages around the importance of workforce in inter-agency partnerships and service coordination to benefit Aboriginal and Torres Strait Islander clients. In particular, the importance of an Aboriginal workforce who are both connected to community and connected to other Aboriginal workers across organisations was considered key to a responsive and culturally safe service system. Putting *‘a face to a name’* and having direct lines of communication were considered essential to effective coordination of services so that workers can *‘ask for favours’* and navigate access to partner services in a timely way. Network activities that brought workers together to build relationships and reflect on service coordination and improvement were considered invaluable. In a service environment where there is high staff turnover, opportunities for newly appointed staff to efficiently build relationships with employees in partner services are critical.

Participants in our study referred to ‘partnering’, ‘collaborating’, ‘coordinating’, ‘communicating’ and forming ‘collaborations’ and ‘alliances’. What we don’t yet know is what level of integration is most beneficial, and possible, from the perspective of service providers in northern Adelaide and with the express intent to strengthen Aboriginal and Torres Strait Islander client outcomes. In the literature, the term ‘partnership’ is used to refer to relationships between services and communities, to refer to connections between service providers, and in referring to connections between governments, services, and communities. We could consider the integration scale proposed by Harris and colleagues [38], that defines inter-organisational integration using an incremental scale from ‘not linked or integrated at all’ through levels of communication, cooperation, coordination, collaboration and partnership to ‘fully linked or integrated’ (p.1672). In this scale, partnership is defined as working ‘together as a formal team with specified responsibilities to achieve common program goals (i.e., have formally identified common goals and areas of responsibility for each organization, usually outlined in a Memorandum of Understanding or other agreement)’ (p. 1672) [38]. We must consider what we are hoping to achieve, and then undertake the necessary steps to achieve it. Further research could clarify the nature of inter-agency relationships in the context of Aboriginal and Torres Strait Islander health and social services, and to define the mechanisms through which agencies can negotiate and achieve their desired level of integration. We are currently engaging service providers in a social network analysis to determine the extent of integration across the health and social service system in northern Adelaide.

The strengths of this study include its focus on a high-need target region, and the engagement of local service providers to garner the perspectives of Aboriginal and Torres Strait Islander and non-Indigenous peoples in understanding place-specific challenges and opportunities. Our Aboriginal Governance Panel promoted rigour through supporting the interpretation of emerging findings to ensure they resonate with the lived experiences of northern Adelaide’s Aboriginal service providers. We applied a rapid translation approach. After sharing early findings related to a lack of workforce networking opportunities, a member of the project’s Aboriginal Governance Panel proactively established the ‘Northern Nungas Network’ with support from the project’s Knowledge Broker. This Network brings together Aboriginal and Torres Strait Islander workforce in northern Adelaide each month and is currently facilitated through goodwill and in-kind support. There have been previous Networks established in northern Adelaide and they have all failed when passionate and motivated workers change roles or leave their organisations. Further research is needed to determine the structural supports and funding mechanisms for such a Network to promote effective and sustainable connection opportunities for Aboriginal and Torres Strait Islander workforce across the region. We will evaluate the impact of the Northern Nungas Network and will rapidly translate other study findings to inform policy and system reform and to promote service coordination. Our social network analysis will quantify intersectoral partnerships in the region and we will consider, with project stakeholders, what level of service integration and coordination is considered beneficial in the context of Aboriginal and Torres Strait Islander health and social services. Future research could also examine the impact of inter-agency partnerships on service system and client outcomes.

## Conclusions

This study has described key leverage points for action on inter-agency partnerships to strengthen service system responses to benefit Aboriginal and Torres Strait Islander communities on Kaurna Country. Sustainable interagency partnerships to benefit Aboriginal and Torres Strait Islander clients across northern Adelaide require system-level reforms to commissioning of services in conjunction with funded network strengthening activities and Aboriginal and Torres Strait Islander practitioner-led approaches to coordination of client-centred care. Like other regions, partnerships in northern Adelaide are challenged by a fragmented, unstable service system with short term contracts and instability in funding mechanisms. We’ve heard that competition between service providers fails Aboriginal and Torres Strait Islander people and wastes resources. Participants in this study proposed that effective interorganisational partnerships to benefit Aboriginal communities in northern Adelaide requires reformed service commissioning processes, aligned intersectoral strategic intentions, sustainable funding, specialisation of services, funded networks, regular network events, and connected Aboriginal and Torres Strait Islander workforce across organisations. Participants also identified the need for Aboriginal and Torres Strait Islander Coordinators to build connections with Aboriginal and Torres Strait Islander clients, determine needs and goals, and coordinate partner services in a staged approach. These findings will be rapidly communicated to policy makers and governments to advocate for system-wide reforms.

## Supplementary Information

Supplementary Material 1.

Supplementary Material 2.

## Data Availability

The datasets generated and analysed during the current study are not publicly available since data was collected under ethical clearances with strict conditions of confidentiality and protection.

## Abbreviations

NGO: Non-government organisations
GOV: Government
ACCO: Aboriginal community-controlled organisations

## Footnotes

1 ‘Nunga lunches’ is a term often used to describe lunches provided to the Aboriginal and Torres Strait Islander community hosted by health or social services.

## References

[1] National Geographic. DNA confirms Aboriginal culture one of Earth’s oldest. 2011. Accessed on June 14th 2023]. https://www.australiangeographic.com.au/news/2011/09/dna-confirms-aboriginal-culture-one-of-earths-oldest/.

[2] Malaspinas A, Westaway M, Muller C, et al. A genomic history of Aboriginal Australia. Nature. 2016;538:207–14. 27654914 10.1038/nature18299 PMC7617037

[3] Anderson I, Robson B, Connolly M, Al-Yaman F, Bjertness E, et al. Indigenous and tribal peoples’ health (the Lancet–Lowitja Institute Global collaboration): a population study. Lancet. 2016;388(10040):131–57. 27108232 10.1016/S0140-6736(16)00345-7

[4] Dawson AP, Warrior E, Pearson O, Boyd MA, Dwyer J, Morey K, Brodie T, Towers K, Waters S, Avila C, Hammond C, Lake KJ, Lampard F, Wanganeen F, Bennell O, Bromley D, Shearing T, Rigney N, Czygan S, Clinch N, Pitson A, BrownA, Howard NJ. Exploring self-determined solutions to service and system challenges to promote social and emotional wellbeing in Aboriginal and Torres Strait Islander people: a qualitative study. Front Public Health. 2023;11:1206371. 10.3389/fpubh.2023.1206371. 10.3389/fpubh.2023.1206371 PMC10556859 37809004

[5] Green A, Abbott P, Luckett T, Davidson P, Delaney J, Delaney P, et al. It’s quite a complex trail for families now’ - provider understanding of access to services for Aboriginal children with a disability. J Child Health Care. 2021;25(2):194–211. 32301329 10.1177/1367493520919305

[6] Riggs E, Block K, Warr D, Gibbs L. Working better together: new approaches for understanding the value and challenges of organizational partnerships. Health Promot Int. 2014;29(4):780–93. 23630133 10.1093/heapro/dat022

[7] Khoury P. Beyond the Biomedical paradigm: the Formation and Development of Indigenous Community-Controlled Health Organizations in Australia International. J Health Serv. 2015;45(3):471–94. 10.1177/0020731415584557 26077856

[8] The Centre of Research Excellence in Aboriginal Chronic Disease Knowledge Translation and Exchange (CREATE). Aboriginal Community Controlled Health Organisations in practice: sharing ways of working from the ACCHO sector. Adelaide: Wardliparingga Aboriginal Health Equity Theme, South Australian Health and Medical Research Institute; 2020.

[9] Campbell M, Hunt J, Scrimgeour D, Davey M, Jones V. Contribution of Aboriginal Community-Controlled Health Services to improving Aboriginal health: an evidence review. Aust Health Rev. 2017;42(2):218–26. 10.1071/AH16149 28263705

[10] Haynes E, Taylor K, Durey A, Bessarab D, Thompson S. Examining the potential contribution of social theory to developing and supporting Australian indigenous-mainstream health service partnerships. Int J Equity Health. 2014;13(1):75. 25242106 10.1186/s12939-014-0075-5 PMC4169641

[11] Australian Bureau of Statistics. Socio-economic indexes for areas (SEIFA), 2016. Canberra: Australian Bureau of Statistics; 2016.

[12] Gibson O, Peterson K, McBride K, et al. South Australian Aboriginal Health Needs and Gaps Report: Northen Adelaide Local Health Network. Adelaide: Wardliparingga Aboriginal Research Unit, South Australian Health and Medical Research Institute; 2017.

[13] Liu D, de Crespigny C, Procter N, Kelly J, Francis H, Posselt M, et al. Comorbidity Action in the North: a study of services for people with comorbid mental health and drug and alcohol disorders in the northern suburbs of Adelaide. Australas Psychiatry. 2016;24(6):592–7. 27406930 10.1177/1039856216657694

[14] Government of South Australia. Northern Adelaide Local Health Network Strategic Plan 2020–2025. Adelaide: Government of South Australia; 2020.

[15] Cairney I, Galletly C, de Crespigny C, Liu D, Moss J, Procter N. Stopping the run-around? A study of services for people with comorbid mental health and substance use disorders in northern Adelaide. Australas Psychiatry. 2015;23(3):233–5. 25783670 10.1177/1039856215576397

[16] National Aboriginal Health Strategy Working Party. The National Aboriginal Health Strategy. Canberra: National Aboriginal Health Strategy Working Party; 1989.

[17] Commonwealth of Australia. National Aboriginal and Torres Strait Islander Health Plan 2021–2031. Canberra: Commonwealth of Australia; 2021.

[18] Taylor K, Thompson S. Closing the (service) gap: exploring partnerships between Aboriginal and mainstream health services. Aust Health Rev. 2011;35(3):297–308. 21871191 10.1071/AH10936

[19] Taylor K, Bessarab D, Hunter L, Thompson S. Aboriginal-mainstream partnerships: exploring the challenges and enhancers of a collaborative service arrangement for Aboriginal clients with substance use issues. BMC Health Serv Res. 2013;10(13):12. 10.1186/1472-6963-13-12 PMC3547814 23305201

[20] Bailey S, Hunt J. Successful partnerships are the key to improving Aboriginal health. NSW Public Health Bull. 2012;23:48–51. 10.1071/NB11057 22697093

[21] Rigney L-R. Internationalization of an indigenous Anticolonial Cultural Critique of Research methodologies: a guide to Indigenist Research Methodology and its principles. Wicazo Sa Rev. 1999;14(2):109–21.

[22] Watson I. Re-centring First Nations Knowledge and places in a Terra Nullius Space. AlterNative: Int J Indigenous Peoples. 2014;10(5):508–20.

[23] Arabena K. Country can’t hear English’: a guide supporting the implementation of cultural determinants of health and wellbeing with Aboriginal and Torres Strait Islander peoples. Riddell’s Creek: Karabena Consulting; 2020.

[24] Wardliparingga Aboriginal Health Equity Unit. South Australian Aboriginal Health Research Accord: Companion Document. Adelaide: South Australian Health and Medical Research Institute; 2014.

[25] Australian Bureau of Statistics. Census 2021, South Australia: Aboriginal and Torres Strait Islander population summary. Canberra: Australian Bureau of Statistics; 2022.

[26] King R, Brown A. Next steps for Aboriginal health research: exploring how research can improve the health and wellbeing of Aboriginal people in South Australia. Adelaide: Aboriginal Health Council of South Australia; 2015.

[27] Lomas J. The in-between world of knowledge brokering. BMJ. 2007;334(7585):129–32. 17235094 10.1136/bmj.39038.593380.AE PMC1779881

[28] Urquhart R, Porter G, Grunfeld E. Reflections on knowledge brokering within a multidisciplinary research team. J Contin Educ Health Prof. 2011;31(4):283–90. 22189993 10.1002/chp.20128

[29] Bessarab D, Ng’andu B. Yarning about Yarning as a legitimate method in Indigenous Research. Int J Crit Indigenous Stud. 2010;3(1):37–50.

[30] The SAHMRI Indigenous Collective. Aboriginal and Torres Strait Islander protocols documen. Adelaide: Wardliparingga Aboriginal Health Equity Unit, South Australian Health and Medical Research Institute; 2018.

[31] Saldana J. The Coding Manual for Qualitative Researchers Los Angeles. SAGE Publications Pty Ltd; 2015.

[32] Graneheim U, Lundman B. Qualitative content analysis in nursing research: concepts, procedures and measures to achieve trustworthiness. Nurse Educ Today. 2004;24(2):105–12. 14769454 10.1016/j.nedt.2003.10.001

[33] Jones J, Barry M. Exploring the relationship between synergy and partnership functioning factors in health promotion partnerships. Health Promot Int. 2011;26:408–20. 21330307 10.1093/heapro/dar002

[34] Finlay SM. Understanding the impacts of the national key performance indicators on Aboriginal Community Controlled Health Organisations [dissertation]. Adelaide: University of South Australia; 2019.

[35] Hope Corbin J, Jones J, Barry M. What makes intersectoral partnerships for health promotion work? A review of the international literature. Health Promot Int. 2018;33(1):4–26. 27506627 10.1093/heapro/daw061 PMC5914378

[36] Brodie T, Pearson O, Cantley L, Cooper P, Westhead S, Brown A, et al. Strengthening approaches to respond to the social and emotional well-being needs of Aboriginal and Torres Strait Islander people: the Cultural pathways Program. Prim Health Care Res Dev. 2021;22:e35. 34184630 10.1017/S1463423621000402 PMC8278791

[37] Gomersall J, Gibson O, Dwyer J, O’Donnell K, Stephenson M, Carter D, et al. What indigenous Australian client’s value about primary health care: a systematic review of qualitative evidence. Aust N Z J Public Health. 2017;41(4):417–23. 28712137 10.1111/1753-6405.12687

[38] Harris J, Luke D, Burke R, Mueller N. Seeing the forest and the trees: using network analysis to develop an organizational blueprint of state tobacco control systems. Soc Sci Med. 2008;67(11):1669–78. 18722038 10.1016/j.socscimed.2008.07.013

