# Supplementary material for: An exploratory qualitative study of inter-agency health and social service partnerships focused on Aboriginal and Torres Strait Islander clients
Source: BMC Health Serv Res. 2024 Dec 18;24:1576. doi: 10.1186/s12913-024-11656-y (PMC11654276; doi:10.1186/s12913-024-11656-y)
Supplement: Supplementary file 1 — Supplementary Material 1. [file 12913_2024_11656_MOESM1_ESM.pdf]

Supplementary File 1

Distress Protocol

Distress

- A participant indicates that they are experiencing a high level of emotional stress; or
- A participant exhibits behaviours which suggest that the discussion is stressful or distressing, such as crying or shaking

Stage 1 response

- **STOP** the interview
- Offer immediate support
- Assess the participant's mental status;
  - ➢ Are you ok?
  - ➢ How are you feeling?
  - ➢ Do you feel safe?
  - ➢ Do you feel able to go on about your day?

Review

- If the participant feels able and willing to carry on, resume the interview
- If the participant is unable to carry on, go to stage 2
- Notify other members of the Research team

Stage 2 Response

- Discontinue the interview
- Ask the participant what support person they would like to seek support from, such as:
  - o their local Employee Assistance Program
  - o a family member
  - o a member of their health care team
- Ask the participant if they would like to contact their support person, or if you can contact them on the participant's behalf.

Follow up

- If the participant consents, the interviewer will contact the participant with a follow up courtesy call to check on their well-being
- Provide the participant with the interviewer's phone number and encourage the participant to call if he/she experiences increased distress in the hours/days following the interview
- Notify other members of the Research Team regarding the enactment of the Distress Protocol

Services to contact for support

Counsellors *[to be updated for each organisation*]	[*Employee Assistance Program counsellor (Indigenous counsellor where available)*]<br><br>*Nominated independent counsellor/s*	T: [*to be updated for each organisation*]
Contact the researcher who will assist in taking appropriate action	[*details removed for privacy purposes*]	T: *[details removed for privacy purposes]*
