# Supplementary material for: An exploratory qualitative study of inter-agency health and social service partnerships focused on Aboriginal and Torres Strait Islander clients
Source: BMC Health Serv Res. 2024 Dec 18;24:1576. doi: 10.1186/s12913-024-11656-y (PMC11654276; doi:10.1186/s12913-024-11656-y)
Supplement: Supplementary file 2 — Supplementary Material 2. [file 12913_2024_11656_MOESM2_ESM.pdf]

Discussion Prompts

1. In what ways do service providers work together in delivering programs and services to the Aboriginal community in Northern Adelaide?

2. Does anyone have a success story they would like to share where you felt that health and social services collaborated effectively in order to support the needs of Aboriginal clients?

3. Are you aware of times when an Aboriginal client has been disadvantaged because of a lack of integration or ineffective partnerships between service providers? What else could have been done to strengthen the connection between services to benefit the client?

4. What factors impact how many other service providers you work with, or the level of integration your service has with other service providers?

5. What are the benefits of effective partnerships – for yourself as a member of the workforce, for your organisation, and for the Aboriginal community?

6. What factors enable or strengthen the partnerships you have with other organisations?

7. What are the challenges to effective partnerships with other service providers?

8. We understand that some Aboriginal clients must navigate numerous health and social service organisations to get their needs met. What strategies could support clients with this navigation, or how could partnerships between organisations be strengthened to support clients to navigate between services?

9. If you had a limitless budget, what strategies would you put in place to strengthen the integration between service providers in Northern Adelaide in order to benefit Aboriginal clients?

10. How would you like implement these strategies? Are there any challenges that need to be overcome?

11. In the next stage of the research, we will be working with community members, service providers and policy makers to codesign a holistic service delivery model that promotes social and emotional wellbeing in the Aboriginal community. This will consider strategies targeted at individuals, at family/peers/kin, at the community, at health and social services and at policy and funding. Is there anything else you would like to add regarding designing strategies to strengthen partnerships between service providers?
